# A Biological Definition and Research Integrated Staging System of Neuronal α‐Synuclein Disease: Research Framework

**DOI:** 10.1002/alz.095053

**Published:** 2025-01-09

**Authors:** Tanya Simuni

**Affiliations:** ^1^ Northwestern University, Chicago, IL USA

## Abstract

The NSD‐ISS Working Group developed a data‐driven approach to: 1) determine a biologic definition for disease; 2) establish a framework for a disease staging platform. NSD is defined by the presence of pathologic n‐asyn (S) assessed by a validated in vivo biomarker and ultimate presence of dopaminergic neuronal dysfunction (D). This biologic definition is independent of the presence of clinical features, or if present, of the specific clinical syndrome. NSD‐ISS integrates these biological anchors (S and D) and degree of functional impairment caused by motor, cognitive or other non‐motor signs. Individuals in stage 0 and 1 are free of clinical signs and can be diagnosed either by presence of fully penetrant pathogenic variants (G) in SNCA gene (Stage 0), n‐asyn alone (Stage 1A) or in combination with dopaminergic neuronal dysfunction (Stage 1B). Stage 2 is characterized by subtle clinical signs but no functional impairment, with either n‐asyn (S) alone (Stage 2A) or in combination with dopaminergic neuronal dysfunction (Stage 2B). Stages 3‐6 require the presence of both biomarkers and stage‐specific increase in severity of functional impairment. We have developed operational definitions and applied staging to PPMI and two interventional studies cohorts.

**Conclusions:**

The NSD‐ISS provides a comprehensive biologically‐based framework essential to advance biologically‐targeted therapeutic development. The NSD‐ISS is expected to evolve as additional biomarkers emerge. We present the first data‐driven application of the NSD‐ISS, highlighting the heterogeneity of the biological stages in a phenotypically homogeneous cohort.

